# The effect of light on follicular development in laying hens

**DOI:** 10.5713/ab.20.0791

**Published:** 2021-04-13

**Authors:** Shi Bin Cheng, Xian Qiang Li, Jia Xiang Wang, Yan Wu, Peng Li, Jin Song Pi

**Affiliations:** 1 College of Animal Science, Yangtze University, Jingzhou, 434103, China; 2 Ostrich Research Institute, Yangtze University, Jingzhou 434103, China; 3 Institute of Animal Husbandry and Veterinary, Hubei Academy of Agricultural Science, Wuhan, 430064, China

**Keywords:** Follicular Development, Light Stress, Ovarian Injury, Oxidative Stress

## Abstract

**Objective:**

The oxidative stress status and changes of chicken ovary tissue after shading were studied, to determine the mechanism of the effect of shading on follicular development.

**Methods:**

Twenty healthy laying hens (40 weeks old) with uniform body weight and the same laying rate were randomly divided into two groups (the shading group and normal light group). In the shading group, the cage was covered to reduce the light intensity inside the cage to 0 without affecting ventilation or food intake. The normal lighting group received no additional treatment. After 7 days of shading, oxidative stress related indicators and gene expression were detected.

**Results:**

Analysis of paraffin and ultrathin sections showed that apoptosis of ovarian granulosa cells (GCs) increased significantly after light shading. Enzyme linked immunosorbent assay results revealed that the levels of total antioxidant capacity, malondialdehyde, superoxide dismutase (SOD), glutathione, catalase (CAT), and other substances in the sera, livers, ovaries, and follicular GCs of laying hens increased significantly after shading for 7 days; and reactive oxygen species (ROS) levels in the livers of laying hens also increased significantly. ROS in the serum, ovarian and GCs also increased. After shading for 7 days, the levels of 8-hydroxy-2 deoxyguanosine in the sera and ovarian tissues of laying hens increased significantly. Cell counting kit-8 detection showed that the proliferation activity of GCs in layer follicles decreased after shading for 7 days; the expression level of the anti-apoptotic gene B-cell lymphoma-2 in ovarian tissue and follicular GCs was significantly reduced, and the expression levels of pro-apoptotic caspase 3 (*casp3*), and *SOD*, glutathione peroxidase 2 (*GPX2*), and *CAT* were all significantly increased.

**Conclusion:**

Oxidative stress induced by shading light has a serious inhibitory effect on follicular development during reproduction in laying hens.

## INTRODUCTION

Stress threatens the stability of the internal environment, and if animals cannot adapt to the environment around them, they often show changes in reproductive function as well as growth and development [1]. Long-term exposure to light stimulation can change the biological rhythm of an organism, which affects almost all physiological processes. Continuous light stress produces important changes to the endocrine system, particularly the pituitary gonadal axis, that manifest as metabolic disorders, premature aging, a shortened life span, increased tumor incidence,abnormal body weight and gonadal development, the level and persistence of gonadotropin-releasing hormone (GnRH) and luteinizing hormone (LH) secretion [2].

Light stress primarily affects birds in two ways. On the one hand, it indirectly regulates the growth and production performance of chickens by regulating the secretion of hormones [3]. Light stimulation is transmitted to the hypothalamus through the optic nerve and acts on photoreceptors to stimulate the secretion of GnRH, which reaches the anterior pituitary through the pituitary portal system. Light also causes the secretion of follicle-stimulating hormone (FSH) and LH. When these hormones reach the ovary of the chicken through the circulation, they promote the development and maturation of follicles [4]. The developing follicles produce estrogen, which maintains secondary sexual characteristics, such as crown redness and phalange opening in chickens. Estrogen, FSH and LH promote the sexual maturity of the chicken and maintain production performance [5]. On the other hand, chickens experience oxidative stress when in a dark environment for an extended period, resulting in production of high levels of oxygen free radicals. Excessive free radicals damage proteins, nucleic acid and other biological macromolecules, disrupting the normal metabolism of the chicken [6]. Oxidative stress can cause protein peroxidation, DNA oxidative damage, cell membrane phospholipid peroxidation, and different degrees of damage to the organism, affecting the normal growth, development, and aging processes of the chicken [7,8].

Oxidative stress caused by any factor has a significant nega tive impact on the development of animal follicles. Studies have shown that oxidative stress induced by hydrogen peroxide (H_2_O_2_) regulates the death of granulosa cells (GCs) through the ROS-JNK-p53 pathway, thereby affecting follicular development [9]. Similarly, H_2_O_2_-mediated oxidative stress induces porcine GC apoptosis and decreases in cell viability, which impair follicular development [10]. Studies have also shown that cigarette smoke condensate (CSC) and benzo(a)pyrene exposure can cause oxidative stress in mouse ovarian follicles, leading to decreased follicular development and premature ovarian failure [11]. In another study, excessive fluoride intake was shown to affect the development of follicular cells by inducing oxidative stress and GC apoptosis in female mice [12]. Oxidative stress can cause decreases in egg production, the number of graded follicles and egg weight, indicating that oxidative stress inhibits follicular development [13]. Previous studies have shown that oxidative stress inhibits follicular development and reduces the feed intake of laying hens, affecting the secretion of gonadotropin in the hypothalamus [14]. It can directly affect ovarian function and inhibit follicular development [15], but the mechanism is not clear. To date, the influence of oxidative stress induced by light stress on chicken ovarian function and follicular development has been rarely reported. Therefore, in this study, we examined the effect of oxidative stress induced by shading exposure on follicular development in laying hens. These findings provide a theoretical basis for alleviating the adverse effects of oxidative stress on the laying performance of laying hens.

## MATERIALS AND METHODS

### Ethics statement

All animal experiments were conducted according to the guidelines established by the Regulations for the Administration of Affairs Concerning Experimental Animals (Ministry of Science and Technology, China, 2004).

### Animals and experimental design

In this study, 20 healthy laying hens (40 weeks old, provided by the Institute of Animal Husbandry and Veterinary Medicine, Hubei Academy of Agricultural Sciences) with uniform body weight and the same laying rate were randomly divided into two groups (the shading group and normal light group) with 10 animals in each group. The hens were kept one per cage in stacked cages at room temperature and fed a basic diet with free access to food and drinking water. In the shading group, a shading cloth was used to cover the cage to reduce the light intensity inside the cage to 0 without affecting ventilation or food intake. The normal lighting group received no additional treatment.

### Sample collection

After 7 days of treatment, the feed intake and egg production of the laying hens in the shading group were significantly reduced. Five chickens were randomly selected from each group for blood collection, and serum was isolated and stored at 4°C. Birds were manually killed by cervical dislocation. The ovaries and livers were collected immediately.

### Paraffin section preparation and hematoxylin-eosin staining

Fresh ovarian blocks (generally no more than 0.5 cm thick) were placed in paraformaldehyde for fixation. Gradient alcohol solutions (5% to 100%) were used for dehydration to gradually remove water from the tissue blocks. Then, the tissue blocks were placed in xylene for clearing. The transparent tissue blocks were placed in melted paraffin for embedding. The embedded wax blocks were cut into thin slices, generally 5 to 8 microns thick, with a microtome, put it hot water and then placed onto the glass slide. The slides were then placed into a 45°C constant temperature oven to dry. The sections were passed through a 100% to 5% alcohol gradient, and then placed in distilled water. The slices that had been put in distilled water were stained with hematoxylin-eosin (HE). The stained sections were dehydrated in pure alcohol and then xylene to make the sections transparent. The transparent slices were sealed with neutral gum, covered with coverslips, and sealed. After the gum dried slightly, a label was attached, and the sliced specimens were observed under an optical microscope (BH-2; Olympus, Tokyo, Japan).

### Detection of ultrastructure

After the chickens were sacrificed, a small piece of the ovary was removed and immediately placed in prechilled fixation solution. After 10 to 30 min, the tissues were finely cut into 1 mm^3^ pieces. The cut ovarian tissue samples were fixed in electron microscopy fixing solution (2.5% glutaraldehyde). After the tissues were rinsed with buffer (0.1 mol/L phosphate buffer), they were fixed with 1% osmium tetroxide fixation solution for 2 h and rinsed with buffer three times. After the tissues were dehydrated in gradient ethanol, they were soaked in 1:1 acetone embedding agent for 1 to 2 h, placed in pure embedding agent overnight, and then polymerized at 37°C for 12 h, 45°C for 12 h, and 60°C for 24 h. The tissues were cut into ultrathin sections with a thickness of 50 to 70 nm using an ultrathin microtome. After staining the ultrathin sections with uranium acetate for 30 min, the sections were imaged under a transmission electron microscope (FEI TecnaiG^2^12, Eindhoven, Netherlands).

### Detection of oxidative stress indices

The levels of FSH, LH, progesterone (P4), and other hormones in the serum, reactive oxygen species (ROS) content, lipid peroxidation (malondialdehyde [MDA] levels) and total antioxidant capacity (T-AOC) in the serum, ovarian tissue, liver tissue and GCs were measured. The activities of the antioxidant enzymes superoxide dismutase (SOD), catalase (CAT), and glutathione (GSH) were detected by enzyme linked immunosorbent assay (ELISA) kits. DNA oxidative damage in ovarian tissue was also detected by an ELISA kit (Hefei Lai Er Biological Technology Co., Ltd., Hefei, China; 2020).

Pretreatment of the cell culture supernatant: Tissue ho mogenates were centrifuged for 20 min (2,000 to 3,000 rpm), and the supernatants were carefully collected. The cell suspension was diluted in phosphate buffered saline (PBS, pH 7.2 to 7.4), and the cell concentration reached approximately 1 million/mL for detection. After repeatedly freezing and thawing, cell integrity was destroyed, and the intracellular components were released. The samples were centrifuged for 20 min (2,000 to 3,000 rpm), and the supernatants were carefully collected. If a precipitate formed during storage, the samples were centrifuged again.

### Granulosa cell isolation and culture

F1 and F2 follicles from the two groups of chickens were collected and placed in ice-cold PBS. The outer blood vessels and connective tissue of the follicles were removed with sterile ophthalmic forceps. The follicles were cut open and quickly squeezed to discharge the yolk. The follicle was slightly swung in PBS with forceps to separate the granular cell layer. The liquid mixed with the granular cell layer was transferred to a 50 mL centrifuge tube, 5 mL of 0.1% type II collagenase was added, and the samples were incubated with shaking at 37°C for 30 min for digestion. Next, 5 mL M199 medium containing 10% fetal bovine serum was added to terminate digestion, the samples were gently pipetted 10 to 20 times and filtered with a 200 mesh cell sieve, the mesh was rinsed with M199 medium, and the filtrate was centrifuged at 1,000 r/min for 10 min. The supernatant was discarded, and the cell pellet was collected and washed twice with culture solution. The cell pellet was resuspended in complete medium (1% penicillin-streptomycin solution + 10% fetal calf serum + M199 medium). Cell counting was performed by trypan blue staining, and the cell viability was above 95%. The cell suspensions were diluted in complete culture solution to a concentration of 1×10^6^ cells/mL. The suspended GCs were added to a cell culture dish and transferred to a cell incubator with 5% CO_2_ and at a constant temperature of at 37°C.

### Cell counting kit-8 assay for detection of the proliferation of granulosa cells

Separated and cultured groups of granular cells were plated in 6-well cell plates, and after the cells were allowed to adhere to the wall for 24 hours, cell counting kit-8 (CCK-8) reagent was added. Absorbance at 450 nm was measured at 24 h, 36 h, 48 h, and 60 h.

### Quantitative polymerase chain reaction analysis

Total RNA was extracted from ovarian tissues using TRIzol. Total cDNA was synthesized using the PrimeScript RT Reagent Kit with gDNA Eraser (Perfect Real Time) (TaKaRa, Dalian, China). Quantitative polymerase chain reaction (Q-PCR) was performed with a LightCycle 480 II instrument (Roche, Basel, Switzerland) in a final volume of 20 μL using Thunderbird SYBR qPCR Mix (TOYOBO, Osaka, Japan). The levels of oxidative stress-related genes, such as B-cell lymphoma-2 (*BCL2*), caspase 3 (*casp3*), *SOD2*, *CAT*, and glutathione peroxidase 2 (*GPX2*), were analyzed, and the data were processed using GraphPad 7.

### Statistical analysis

All data represent the mean±standard deviation of at least three independent experiments. Statistical differences were determined by Student’s t-test or one-way analysis of variance with GraphPad Prism 5. The results were considered statistically significant at p<0.05.

## RESULTS

### Histomorphological observation

**Figure 1:**
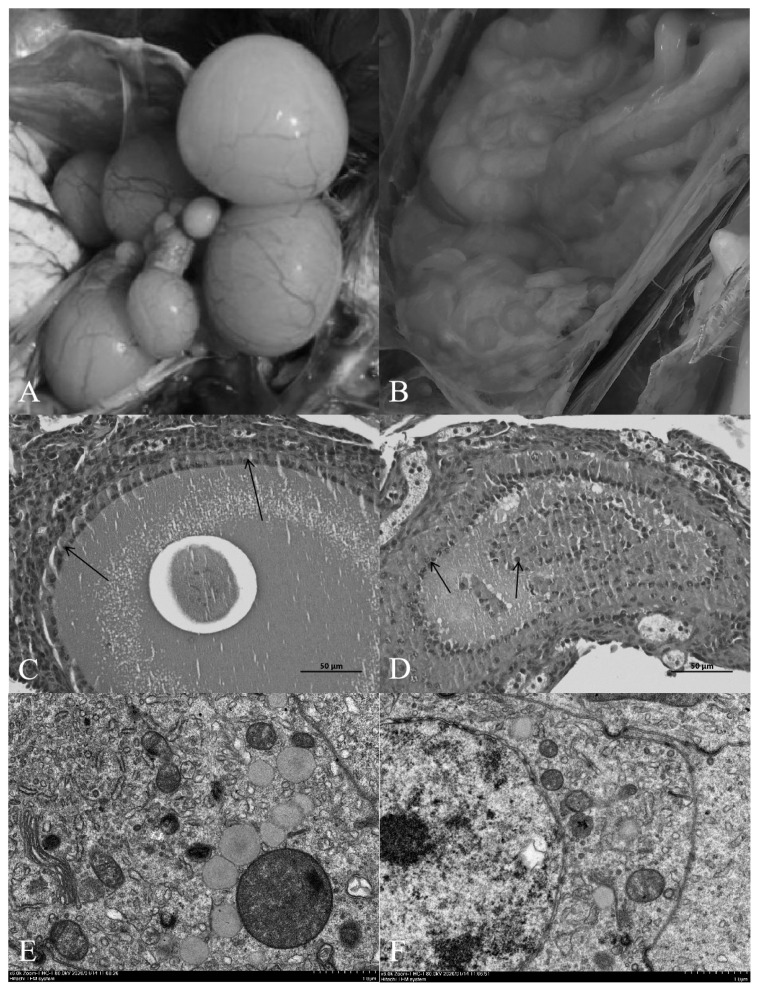
(A) and (B) correspond to the anatomical maps of ovarian tissues of normal lighting group and shading group respectively. (C) and (D) correspond to hematoxylin-eosin (HE) stained sections of layer ovarian tissue in the normal lighting group and shading group respectively. Granular cells (arrows) were observed in both groups (Bar, 50 μm). (E) and (F) correspond to the transmission electron microscope slices of the ovarian tissue of laying hens in the normal illumination group and the shading group respectively; (E, F) Scale bar, 1.0 μm.

Compared with the normal light group, the follicles in the ovary of the shading group showed severe atresia, and the normal grade follicles were almost invisible (Figure 1A, 1B). The HE results showed that the follicular wall layers of the normal group were complete, the basal membrane was tightly bound to the granulosa layer, and the granulosa layer cells were tightly bound to each other. There were no detached GCs in the follicular cavity, the cytoplasm and nuclear chromatin were uniformly stained, and the nucleus was in the center of the cell (Figure 1C). In the group shaded from light, the outer layer of the atretic follicle wall was intact, the basal membrane had degenerated, and the granular layer was separated to varying degrees. Cells in the granular layer were loosely bound to each other, and some or all the GCs had shed into the follicular cavity. The nuclei of GCs were hyperchromatic or fragmented into atretic bodies (Figure 1D).

Transmission electron microscopy revealed that the nu cleoli of normal ovarian GCs were obvious and easy to detect. The euchromatin and heterochromatin were evenly distributed in the nucleus, and euchromatin was abundant. The nuclear membrane was complete and smooth, and there were many mitochondria, rough endoplasmic reticulum, Golgi apparatus and other organelles in the cytoplasm (Figure 1E). Chromatin condensation and aggregation towards the edge of the nucleus were observed in GCs in the light-shaded group, resulting in increased nuclear electron density, nuclear membrane shrinkage and vacuoles in the cytoplasm. Microscopy showed that apoptotic bodies had formed after fragmentation of the cells, which were the remnants of apoptotic cells and formed by partially highly condensed chromatin wrapped in the plasma membrane (Figure 1F).

### Oxidative stress index detection

After shading treatment, FSH, LH, and P4 levels in the serum, ROS content, lipid peroxidation (MDA activity), and the activities of the antioxidant enzymes T-AOC, SOD, CAT, and GSH in the serum, liver tissue ovarian tissue and granular cells were detected.

The results demonstrated that the serum FSH and LH levels of laying hens in the shading group were significantly lower than those in the normal light group on day 7 (p<0.05) and that P4 levels were significantly increased (p<0.01) (Table 1). Furthermore, MDA levels in the sera, livers, ovaries, and follicular GCs of laying hens in the shading group were significantly higher than those of laying hens in the normal light group (p<0.05). On the 7th day, SOD and T-AOC levels in the sera, livers and follicular GCs of the hens were significantly higher in the shading group than in the normal light group (p<0.05). Among these changes, the change in T-AOC levels in follicular GCs was extremely significant (p<0.01), but there was no significant difference in the levels of SOD or T-AOC in the ovaries of laying hens (p>0.05). On the 7th day, the levels of GSH and CAT in the serum, liver, ovary, and follicular GCs were significantly increased in the shading group compared with the normal light group (p<0.05), and the change in CAT levels in the follicular GCs was extremely significant (p<0.01). After 7 days of shading treatment, ROS content in the liver tissues of laying hens was significantly higher than that in the liver tissues of laying hens in the normal light group (p<0.01). ROS content in the serum, ovaries and follicular GCs also showed an upward trend, but the difference was not statistically significant (p>0.05) (Table 2).

### DNA oxidative damage detection

An ELISA kit was used to detect 8-hydroxy-2 deoxyguanosine (8-OHdG) content to reflect DNA oxidative damage (Table 3). The levels of 8-OHdG in the sera and ovarian tissues of laying hens in the shading group were significantly lower than those in the sera and ovarian tissues of laying hens in the normal light group on day 7 (p<0.05).

### Detection of proliferation of granulosa cells

**Figure 2:**
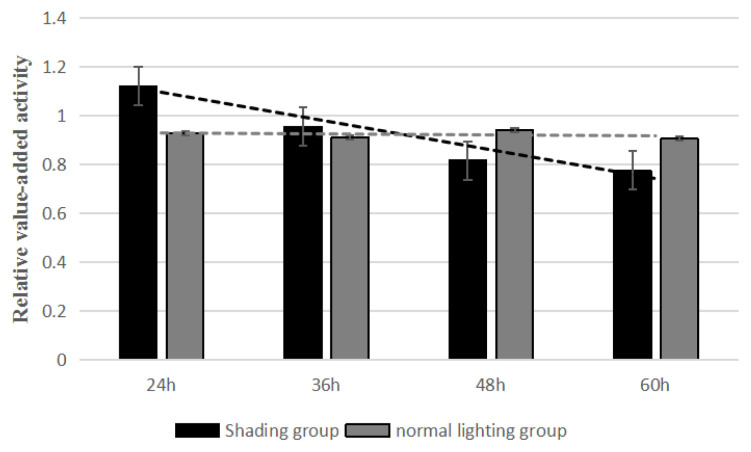
The effect of shading treatment on the proliferation activity of layer follicle granulosa cells.

The results of CCK-8 test showed that the relative proliferation activities of the shading group were 1.122, 0.956, 0.818, and 0.776 for 24, 36, 48, and 60 h and those of the control group were 0.929, 0.911, 0.942, and 0.907. Compared with the control group, the GC proliferation in the shading group was significantly decreased from 0 to 60 h (Figure 2). This indicated that the proliferation of layer GCs was decreased when shading treatment.

### Oxidative stress-related genes expression analysis

**Figure 3:**
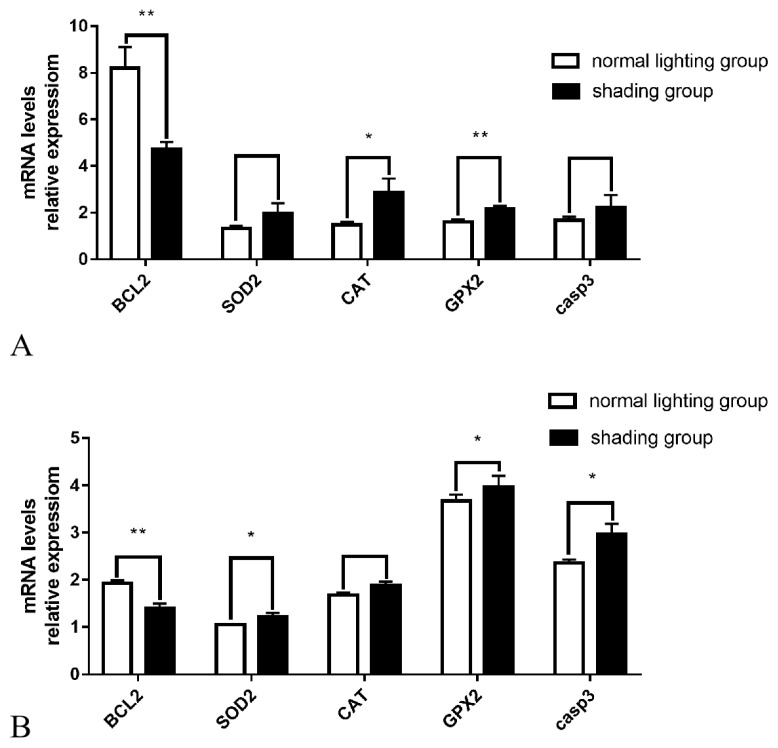
Effect of shading treatment on the expression level of related genes in ovarian tissue and granulosa cells. (A), (B) correspond to ovarian tissue and granulosa cells. Compared with the control group, * p<0.05, ** p<0.01.

The Q-PCR results showed that after 7 days of shading treatment, the expression level of the anti-apoptotic gene *BCL2* in ovarian tissue was significantly reduced (p<0.01), the gene expression level of the antioxidant enzyme *GPX2* was significantly increased (p<0.01), the gene expression level of the antioxidant enzyme *CAT* was significantly increased (p<0.05), and the gene expression levels of *casp3*, which promotes apoptosis, and the antioxidant enzyme *SOD* also exhibited an upward trend (p>0.05) (Figure 3A). The expression levels of the anti-apoptotic gene *BCL2* in follicular GCs was significantly decreased (p<0.01), the gene expression levels of pro-apoptotic *casp3* and the antioxidant enzymes *SOD* and *GPX2* were significantly increased (p<0.05), and antioxidant enzyme *CAT* gene expression levels also showed an upward trend (p>0.05) (Figure 3B).

## DISCUSSION

A lack of light in poultry causes oxidative stress, which subsequently affects ovarian function by inducing apoptosis of follicular GCs. When oxidative stress occurs, the activity of antioxidant enzymes in follicle granule cells decreases, oxidative damage occurs to intracellular proteins and DNA, the energy supply to the mitochondria of the cells is disrupted, and the structure and function of the cell membrane are changed. Eventually GCs systematically die.

In this study, shading treatment was used to induce oxi dative stress in hens. After shading exposure, the levels of FSH and LH in the serum were decreased, and the P4 level was significantly increased. Changes in the light cycle may regulate seasonal reproduction in birds by regulating the synthesis and release of hypothalamic and pituitary secretions [14]. In a study, 24 h of light exposure led to a reduction in the total number of ovarian follicles in mice and downregulation of circadian rhythm-related genes within a short period of time (less than 2 weeks), and changes in serum sex hormone levels were also be detected after 24 h, including increased concentrations of LH, FSH, and E2 (estradiol) and a decreased concentration of PROG (progesterone) [15]. Studies have shown that hens exposed to light for an extended period exhibit higher plasma levels of FSH and LH before laying eggs and that the expression levels of *FSH* and *LH* genes are increased [4]. Light induces expression of several genes, playing an important role in regulating the secretion of hormones related to the pituitary-gonad axis and sexual and reproductive functions in animals and humans [16]. In a study on the effect of the photoperiod on ovarian morphology, reproductive hormone secretion and hormone receptor mRNA expression in ducklings, higher serum FSH, LH, and progesterone levels and ovarian follicle hormone receptor mRNA expression levels were observed when the photoperiod was longer than 10 h [17]. In estrogen-promoted prepubertal hen maturation responses to photostimulated FSH and LH, plasma FSH and LH concentrations increase after photostimulation [18]. Blue and green monochromatic light promotes egg production by stimulating gonad secretion and upregulating the expression of estrogen and progesterone receptors [19]. When the light cycle is increased, neuroendocrine system regulation is engaged, causing reproductive activities to last longer, and improving egg production performance [20]. These studies have shown that prolonged light exposure affects the secretion of some hormones in poultry, and these results were supported by this study. A lack of light leads to a reduced number of follicles in poultry, changes the secretion of related hormones, and slows the development of follicles, ultimately leading to a decrease egg laying in hens.

The occurrence of oxidative stress is accompanied by changes in the body’s antioxidant enzyme activity. In this study, we found that after 7 days of shading exposure, the levels of T-AOC, MDA, SOD, GSH, CAT, and other substances in the sera, livers, ovaries, and follicular GCs of laying hens were significantly increased and that ROS levels in the livers of laying hens were significantly increased. ROS levels in the serum and ovarian and GC ovarian tissues also exhibited an upward trend, and the mRNA expression levels of *SOD*, *CAT*, and *GPX2* gene were significantly increased. 3-Nitropropionic acid induces ovarian oxidative stress and damages follicles in mice [21]. Furthermore, oxidative stress and the mRNA expression of CAT and SOD2 are increased after deltamethrin treatment [22]. After treatment of in porcine ovarian GCs with diazinon (DZN) for 48 hours, cellular ROS levels increase significantly, and the mRNA levels of the antioxidant enzymes CAT, SOD2, PRDX6, and GPX1 increase significantly [23]. Treatment of bovine GCs with H_2_O_2_ causes the accumulation of ROS, decreases the activity of mitochondria, increases the expression of Nrf2 and its downstream antioxidant genes (at the mRNA and protein levels), alters cell cycle transition and induces apoptosis [24]. Fluoride-induced oxidative stress in mice causes ovarian the mRNA expression levels of antioxidant enzymes (including CAT, GSH-Px1, and SOD1) to be significantly downregulated, while ROS, MDA, and T-NO levels in the ovary are significantly increased and SOD, GSH-Px, and T-AOC activities are significantly reduced [25]. The results of these studies are basically consistent with the findings of this study, and the slight differences might be partly due to oxidative stress caused by different stimuli. These results indicate that oxidative stress does indeed cause changes in the body’s antioxidant system.

Loss of light exposure in birds induces oxidative stress, which can cause ovarian damage by affecting apoptosis of GCs. This study observed that after 7 days of shading exposure, and the expression level of apoptosis-promoting gene casp3 in GCs increased significantly. There is no significant difference in the expression level of casp3 in ovarian tissues, which may be due to the lack of expression of apoptosis-promoting genes in other cells in ovarian tissues. In the follicles of 3-nitropropionic mice with acid-induced oxidative stress, oxidative stress significantly reduces the ratio of BCL2 to Bax [21]. The gene expression levels of *CAT* and *GPX* in goose granule cells treated with 3-NPA are significantly increased, *BCL2* gene expression levels are significantly reduced, and *Bax*, *p53*, and *Caspase 3* gene expression levels are significantly increased [26]. Studies have also found that oxidative stress occurs in mouse oocytes after deltamethrin treatment, resulting in increased CAT and SOD2 mRNA expression and DNA damage, increased caspase-3 and Bax mRNA expression levels and a significant reduction in BCL-xl mRNA expression [22]. Some studies have found that elevation of ROS expression levels in GCs in patients with polycystic ovary syndrome significantly induces apoptosis and thereby affect oocyte quality [27]. In that study, it was found that miR-145 protects GCs from H_2_O_2_-induced apoptosis by targeting Kruppel like factor 4 (*KLF4*), which promotes H_2_O_2_-induced GC apoptosis through the BAX/BCL2 pathway [28]. After 90 days of fluoride treatment, the relative expression levels of caspase-3, caspase-9, and Bax mRNA in the ovaries of mice exposed to oxidative stress are significantly upregulated, and expression levels of BCL2 are significantly downregulated [25]. Upregulation of FoxO1 by oxidative stress leads to apoptosis of mouse GCs and ultimately to atresia of mouse follicles [29]. In studies of oxidative stress-induced apoptosis in granular cells involving JNK, p53, and Puma, ROS, including H_2_O_2_, were shown to play a key role in the apoptosis of granular cells [9]. Studies have shown that DZN induces apoptosis of mouse GCs by inhibiting the PI3K-AKT pathway, which is related to DNA damage and oxidative stress [23].

Taken together, these findings indicate that oxidative stress is an important inducer of atresia of ovarian follicles. On the one hand, the occurrence of oxidative stress is accompanied by changes in DNA oxidative damage and antioxidant systems, which affect the secretion of hypothalamic-pituitary-gonadal axis-related hormones and follicular development, indirectly regulating avian ovarian function. On the other hand, oxidative stress induces the expression of downstream apoptosis-related genes by affecting the expression and transcription of transcription factors in follicular GCs, leading to apoptosis of GCs and follicular atresia, which in turn causes ovarian dysfunction. In poultry production, reasonable regulation of light and a reduction in light stress are necessary for the healthy development of poultry.

## Figures and Tables

**Table 1:** The effect of shading on follicle-stimulating hormone, luteinizing hormone, and progesterone in serum

Item	Normal lighting group	Shading group	p-value
FSH (ng/mL)	134.77±13.04^b^	114.69±7.05^a^	0.032
LH (mIU/mL)	7.00±0.25^b^	6.11±0.46^a^	0.015
P_4_ (ng/mL)	10.32±2.12^b^	15.95±3.63^a^	0.0087

The data are presented as the mean value±standard deviation.

FSH, follicle-stimulating hormone; LH, luteinizing hormone; P4, progesterone.

a,b Mean values without the same letters are significantly different (p<0.05).

**Table 2:** The effect of shading on MDA, SOD, T-AOC, GSH, CAT, and ROS content in the serum, liver, ovary and follicle granulosa cells

Items		Normal lighting group	Shading group	p-value
MDA content (nmol/mL)	Serum	6.25±0.33^b^	7.27±0.21^a^	0.025
	Liver	7.52±0.77^b^	8.01±0.42^a^	0.042
	Ovary	7.34±0.52^b^	9.22±1.56^a^	0.011
	Granulosa cells	7.82±0.36^b^	11.91±0.22^a^	0.049
SOD content (ng/mL)	Serum	5.58±0.46^b^	6.05±0.26^a^	0.011
	Liver	5.86±0.66^b^	7.31±0.96^a^	0.012
	Ovary	6.02±0.11	5.40±1.26	0.279
	Granulosa cells	7.25±0.89^b^	11.32±2.96^a^	0.017
T-AOC content (U/mL)	Serum	12.34±0.73^b^	13.09±1.24^a^	0.027
	Liver	12.67±0.67^b^	15.03±1.46^a^	0.023
	Ovary	13.77±0.78	15.95±2.73	0.280
	Granulosa cells	15.85±0.72^b^	24.38±6.13^a^	0.004
GSH content (ng/mL)	Serum	4.31±0.23^b^	4.53±0.40^a^	0.012
	Liver	4.73±0.18^b^	5.35±0.46^a^	0.040
	Ovary	3.94±0.58^b^	5.22±1.05^a^	0.036
	Granulosa cells	4.90±0.74^b^	7.25±1.74^a^	0.042
CAT content (pg/mL)	Serum	367.63±4.49^b^	393.03±30.01^a^	0.029
	Liver	403.82±22.14^b^	460.83±53.97^a^	0.039
	Ovary	431.12±30.60^b^	484.08±107.18^a^	0.011
	Granulosa cells	523.88±44.95^b^	743.95±221.24^a^	0.007
ROS content (IU/mL)	Serum	555.42±45.11	595.93±45.10	0.126
	Liver	596.25±29.65^b^	654.96±10.08^a^	0.0064
	Ovary	714.62±22.43	791.54±73.27	0.079
	Granulosa cells	657.99±6.45	670.69±4.98	0.079

The data are presented as the mean value±standard deviation.

MDA, malondialdehyde; SOD, superoxide dismutase; T-AOC, total antioxidant capacity; GSH, glutathione; CAT, catalase; ROS, reactive oxygen species.

a,b Mean values without the same letters are significantly different (p<0.05).

**Table 3:** Effect of shading on 8-hydroxy-2 deoxyguanosine levels (DNA oxidative damage) (ng/mL) in the ovarian tissues of laying hens

Items	Normal lighting group	Shading group	p-value
Serum	37.85±4.45^b^	52.43±2.84^a^	0.012
Ovarian tissue	46.01±1.10^b^	57.64±5.40^a^	0.047

The data are presented as the mean value±standard deviation.

a,b Mean values without the same letters are significantly different (p<0.05).
